# The epidemiology of uterine fibroids: global disease burden from 1990 to 2021 and future trend predictions

**DOI:** 10.3389/frph.2025.1629834

**Published:** 2025-09-18

**Authors:** Chan Wu, Ling Zhou, Ruilin Chen, Huiling Li, Jian Li, Feifei Guo, Rong Li, Huaijun Zhou, Jingjing Huang

**Affiliations:** ^1^ Department of Gynecology, Nanjing Drum Tower Hospital, Affiliated Hospital of Medical School, Nanjing University, Nanjing, China; ^2^ Department of Obstetrics and Gynecology, Peking University People’s Hospital, Beijing, China; ^3^ Department of Gynecology, Nanjing Drum Tower Hospital Clinical College of Nanjing University of Chinese Medicine, Nanjing, China

**Keywords:** uterine fibroids, global burden of disease, health inequality, Bayesian age-period-cohort (BAPC), prediction

## Abstract

**Background:**

Uterine fibroids (UF) are the most common benign tumors of the female reproductive system, imposing a significant health burden. A comprehensive understanding of their global, regional, and national burden is essential for targeted public health planning. This study aimed to analyze the spatiotemporal trends of UF burden from 1990 to 2021 and project future trends to 2036.

**Methods:**

Data on the incidence, prevalence, and disability-adjusted life years (DALYs) of UF from 1990 to 2021 were extracted from the Global Burden of Disease (GBD) 2021 study, covering 204 countries and territories. We analyzed trends using estimated annual percentage changes (EAPC) for age-standardized rates. Socio-demographic Index (SDI) was used to assess the association between development level and disease burden. The Slope Index of Inequality (SII) and concentration index were employed to quantify health inequalities. A Bayesian age-period-cohort (BAPC) model was used to project the burden to 2036.

**Results:**

Between 1990 and 2021, the global ASIR of UF rose from 234.36 (95%UI: 171.06, 309.92) to 250.93 (183.44, 330.94) per 100,000 [EAPC 0.24 (0.23, 0.25)]. The ASPR increased from 2799.88 (2133.46, 3650.54) to 2841.07 (2164.43, 3682.27) [EAPC 0.04 (0.03, 0.06)]. DALYs grew from 81,142 (57,125, 111,989) to 142,885 (102,183, 192,988), while ASDR showed little change, from 3.48 (2.46, 4.77) to 3.39 (2.43, 4.59). Regional analysis indicated that South Asia exhibited the highest incidence and prevalence of UF, whereas Oceania and Australia experienced a lower burden. Analysis of health inequality revealed a shift in the burden of UF incidence and prevalence from high to low Socio-Demographic Index (SDI) countries between 1990 and 2021, indicating a reduction in health inequality. Future predictions from the BAPC model indicate that both ASIR and ASPR are expected to continue to rise, while ASDR is likely to decline.

**Conclusions:**

From 1990 to 2021, the global incidence of UF has steadily risen, with South Asia experiencing the greatest impact. Despite the stabilization of ASDR, the rise in ASIR and ASPR remains a significant public health challenge worldwide. Health inequality analysis indicates that the burden of UF is shifting toward low SDI countries. Future prevention and treatment strategies for UF should focus on middle- and low-income countries, specifically by implementing targeted screening programs, investing in low-cost diagnostic tools, and launching public health awareness campaigns. Global public health cooperation, along with early diagnosis and treatment strategies for UF, will be crucial in reducing the disease burden.

## 1 Introduction

Uterine fibroids (UF), which are prevalent benign tumors in the female reproductive system, consist mainly of smooth muscle cells and fibrous connective tissue. Epidemiological studies indicate that UF are highly prevalent among women of reproductive age, with a notably higher incidence in women over the age of 40. Racial factors significantly influence the incidence of UF (1). Studies have consistently demonstrated a higher incidence of UF in Black women compared with White women (2, 3).

UF significantly affect women's health, particularly by causing menstrual irregularities such as heavy, prolonged, or irregular bleeding, which may substantially impair quality of life (4). Additionally, UF can cause chronic pelvic pain, dyspareunia (painful intercourse), and bladder pressure, further exacerbating the patient's daily burden. Furthermore, UF are associated with infertility, particularly when the fibroids are submucosal or alter the shape of the uterine cavity, potentially impairing reproductive ability. UF during pregnancy can result in adverse outcomes, including preterm birth, abnormal fetal positioning, and postpartum hemorrhage (5, 6).

The consumption of healthcare resources due to UF is also a significant issue (7). Healthcare systems must allocate significant resources for diagnosing and treating UF due to their high incidence and varied treatment options (8). The treatment options for uterine fibroids primarily include expectant management, pharmacological therapy, interventional procedures, and surgery. Although hysterectomy offers definitive removal of lesions, it results in the permanent loss of fertility. Uterus-preserving surgical and interventional approaches, while avoiding hysterectomy, are frequently associated with long-term impairment of reproductive function and a higher risk of recurrence (9). Pharmacological treatment is not suitable for women actively seeking pregnancy, as its efficacy is usually limited to the treatment period, and its long-term safety remains uncertain. For instance, gonadotropin-releasing hormone agonists (GnRHa) may lead to irreversible bone loss and osteoporosis, whereas ulipristal acetate has been reported to be associated with severe liver injury (10). Therefore, current treatment strategies remain limited in terms of efficacy, safety, and preservation of fertility (3, 11, 12). Consequently, UF management requires not only addressing patient health and quality of life but also poses substantial challenges for healthcare resource allocation and utilization.

The social and medical costs associated with UF have become a significant public health concern. Evidence indicates that the economic burden of UF includes not only direct medical expenses but also substantial indirect costs, such as loss of work productivity and obstetric complications (13). A systematic review covering studies worldwide reported that the annual per-patient total cost of UF ranged from USD 11,717 to 25,023, with an incremental annual cost of USD 2,200 to 15,952 compared with control populations (14). In the United States, the socioeconomic burden of UF was estimated at USD 5.9–34.4 billion in 2012, which had risen to USD 41.4 billion by 2022 (15). When costs associated with magnetic resonance-guided focused ultrasound surgery (MRgFUS) and infertility were included, the total burden increased to USD 42.2 billion. These findings underscore that UF imposes not only a clinical challenge but also a substantial economic and societal burden worldwide.

Both the incidence and prevalence of UF vary significantly across racial and regional groups. According to a study in the United States, the incidence of UF varies significantly by age and race, with Black women experiencing a notably higher prevalence (16, 17). Furthermore, a study exploring UF prevalence in Latina women highlighted that, despite known racial differences, data across diverse ethnic groups remain limited (18). An Australian study offered initial estimates of the incidence and prevalence of UF in reproductive-age women (19). The study identified that 7.3% of women aged 45–49 were diagnosed with UF, with the highest incidence occurring in those aged 40–44. Current epidemiological studies on UF have mainly focused on the national or regional level, whereas research at the global scale remains limited. To date, only analytical studies based on the 2019 Global Burden of Disease (GBD) data have investigated the global epidemiology of UF (20, 21). Moreover, no predictive analyses of future trends, assessments of health inequalities, or cluster analyses have been conducted at the global level, and no epidemiological studies utilizing the 2021 GBD data are currently available. These gaps underscore the urgent need for updated and comprehensive global epidemiological analyses of UF.

Given the substantial impact of UF on health, economics, and public health, together with the paucity of up-to-date research, systematically analyzing the global disease burden of UF and predicting future trends is of great importance. Against this background, the present study aims to investigate the burden and temporal trends of UF from 1990 to 2021 using data from the 2021 GBD study. The findings are expected to provide evidence to guide global and national strategies for UF prevention and management and to inform future research directions.

## 2 Materials and methods

### 2.1 Data source

The GBD database is a major global health platform offering comprehensive data on numerous diseases and injuries. It uses standardized statistical methods and contributions from global health experts to comprehensively evaluate the burden of diseases. This study utilized data from the GBD 2021 study to evaluate the global, regional, and national burden and trends of UF from 1990 to 2021. The GBD dataset was selected as the primary data source for several key reasons. First, its comprehensive and standardized methodology allows for robust comparisons of disease burden across different geographic locations and time periods, which is essential for a global epidemiological analysis. Second, the GBD provides estimates for countries and regions where local data may be scarce or unavailable, filling critical knowledge gaps.

However, the use of GBD data has inherent limitations that must be acknowledged. The quality and availability of primary data vary significantly across regions, with low- and middle-income countries often having less robust surveillance and reporting systems. The GBD framework addresses this through statistical modeling, including covariate-driven models and spatio-temporal Gaussian process regression (ST-GPR), to estimate missing data and correct for inconsistencies. This modeling can introduce uncertainty and potential biases, particularly in data-sparse regions where estimates are heavily reliant on covariates rather than direct measurement. For UF, which are often asymptomatic and underdiagnosed, the reliance on administrative and survey data may lead to an underestimation of the true prevalence and incidence. This study interprets all findings in the context of these strengths and weaknesses.

### 2.2 Data extraction and case definition

GBD employs a range of data sources, including census data, disease registry databases, clinical data, health surveys, and research, as well as various national and regional statistics. The GBD methodology for non-fatal diseases like uterine fibroids relies on a comprehensive data synthesis process. For UF, case definitions were based on the International Classification of Diseases (ICD), specifically ICD−10 codes (D25.0, D25.1, D25.2, D25.9). The primary data sources included published literature, survey data, and hospital administrative data. The prevalence of UF was estimated using DisMod-MR 2.1, a Bayesian meta-regression tool that synthesizes diverse data sources to produce consistent estimates of incidence, prevalence, and remission. This model enforces consistency between epidemiological parameters. ST-GPR was then used to interpolate data for locations and years with missing information, borrowing strength from neighboring locations and time points to generate a complete and comparable set of estimates. The study indicators chosen were “Incidence,” “Prevalence,” “Deaths,” “DALYs (Disability-Adjusted Life Years),” “YLDs (Years Lived with Disability),” and “YLLs (Years of Life Lost)”. The data was further collected and analyzed based on different geographic locations, socio-economic development levels, and age groups.

### 2.3 Statistical analysis

This study utilized the Estimated Annual Percentage Change (EAPC) to evaluate temporal trends in the burden of UF from 1990 to 2021. EAPC is a key statistical measure for assessing the rate of change in disease burden and reflects the average annual change in UF over a specific period. The method calculates the slope (*β*) by applying a logarithmic transformation to the age-standardized rates (ASR) for each year using a linear regression model. The formula for calculating EAPC is as follows:

$$\mathrm{EAPC}=(e^{\beta }-1)\times 100$$

where *β* is the slope parameter from the regression model. Calculating the EAPC allows for a quantitative analysis of global, regional, and national trends in the incidence and mortality of UF. When the 95% confidence interval (CI) for EAPC is entirely positive, it indicates a significant increase in disease burden; when the CI is entirely negative, it indicates a significant decrease in disease burden.

To quantify socioeconomic inequality in the burden of UF across countries, we calculated the Slope Index of Inequality (SII) and the concentration index. The SII represents the absolute difference in health outcomes between the highest and lowest ends of the socioeconomic spectrum. It is derived from a regression of the health outcome on the cumulative proportion of the population ranked by SDI. A positive SII indicates that the burden is higher in countries with higher SDI. The concentration index is a relative measure of inequality. It is calculated as twice the area between the concentration curve (which plots the cumulative proportion of the health outcome against the cumulative proportion of the population ranked by SDI) and the line of equality. The concentration index ranges from −1 to +1, where a positive value indicates that the burden is concentrated among the higher SDI groups, a negative value indicates concentration among the lower SDI groups, and zero signifies perfect equality.

This study employed health inequality analysis to evaluate the disparities in UF burden among groups with varying Socio-Demographic Index (SDI) levels, utilizing the Slope Index of Inequality (SII) and the Concentration Index as indicators. The SII quantifies the absolute disparity in health indicators between the highest and lowest SDI groups. The SII calculation is based on relative rankings and is obtained using a robust regression model. This model minimizes outlier sensitivity and bias from data heterogeneity or extreme values, enhancing the accuracy of health inequality assessments. A higher SII value indicates a larger health burden gap between groups, typically suggesting that lower SDI groups bear a larger health burden. In this study, we calculated the SII values for incidence, prevalence, and DALYs rates to assess the trends in these indicators across different SDI levels. The Concentration Index is used to measure the concentration of health indicators within the distribution of socio-economic status, reflecting the relative distribution of the study indicators across different socio-economic groups. The Concentration Index, ranging from [−1, 1], is derived by integrating the cumulative proportion of DALYs with the cumulative population distribution and SDI rankings beneath the Lorenz curve. Positive values indicate that the health indicator is more concentrated in high SDI groups, while negative values suggest a greater concentration in low SDI groups. We used the Concentration Index to analyze the distribution of incidence, prevalence, and DALYs across different SDI levels.

Hierarchical clustering was utilized to examine regional similarities in the changes in UF burden. We calculated the Euclidean distances between the EAPC values of age-standardized incidence rate (ASIR) and age-standardized DALYs rate (ASDR) for each region, resulting in a distance matrix. The maximum distance method was then used to perform hierarchical clustering on this data. Countries with similar burden trends were grouped together, allowing us to identify regional patterns in the burden of UF. The results of the clustering analysis were visualized through dendrograms and scatter plots, providing a more intuitive representation of the differences and trends in the burden of UF across regions and countries.

In this study, we employed the Bayesian Age-Period-Cohort (BAPC) model to forecast the global burden of uterine fibroids over the next 15 years. The BAPC model is a regression model based on the Bayesian statistical framework, widely used for analyzing disease trends, especially suitable for disease burden studies involving multidimensional data such as age, period, and cohort effects. The BAPC model was chosen over alternatives like Autoregressive Integrated Moving Average (ARIMA) because it can effectively disentangle the distinct influences of age (biological aging), period (external factors affecting all age groups at a specific time), and cohort (generational effects), which are critical for understanding long-term epidemiological transitions. While ARIMA models are effective for time-series forecasting, they do not differentiate these underlying drivers, making BAPC more mechanistically informative for population health data. This model decomposes key health indicators, such as ASIR, age-standardized prevalence rate (ASPR), and ASDR, into these three main components, thus enabling a deeper understanding of the dynamic changes in disease burden. The core hypothesis of the BAPC model is that the incidence of a disease is influenced by the interaction of age, period, and birth cohort effects. To implement this, we assigned appropriate prior distributions to each of these effects. The age effect was modeled as a smooth normal distribution, the period effect as a multinomial distribution, and the cohort effect as a distribution with long-term cyclical fluctuations. Weakly informative priors were selected to minimize their influence on the posterior distribution, allowing the data to primarily drive the results. Under the Bayesian framework, prior distributions were combined with the data, and the model was fitted using the Integrated Nested Laplace Approximations (INLA) method, a computationally efficient alternative to Markov Chain Monte Carlo (MCMC) for fitting Bayesian models. To ensure the validity and robustness of the BAPC model, we performed posterior predictive checks after fitting the model, verifying its appropriateness and stability. Model convergence was assessed by examining trace plots and ensuring the Gelman-Rubin statistic (R-hat) was below 1.1.

Data analysis and modeling in this study were performed using R (version 4.5.1), along with several commonly used R packages. All models and statistical analyses underwent rigorous diagnostics to ensure the convergence and accuracy of the models.

### 2.4 Ethical statement

Ethical approval for the Global Burden of Disease (GBD) study was obtained from the Institutional Review Board of the University of Washington. The present analysis is based on secondary use of aggregated, publicly available, and de-identified GBD data; therefore, no additional ethical approval or informed consent was required.

## 3 Results

### 3.1 Global burden of UF from 1990 to 2021

**Figure 1:**
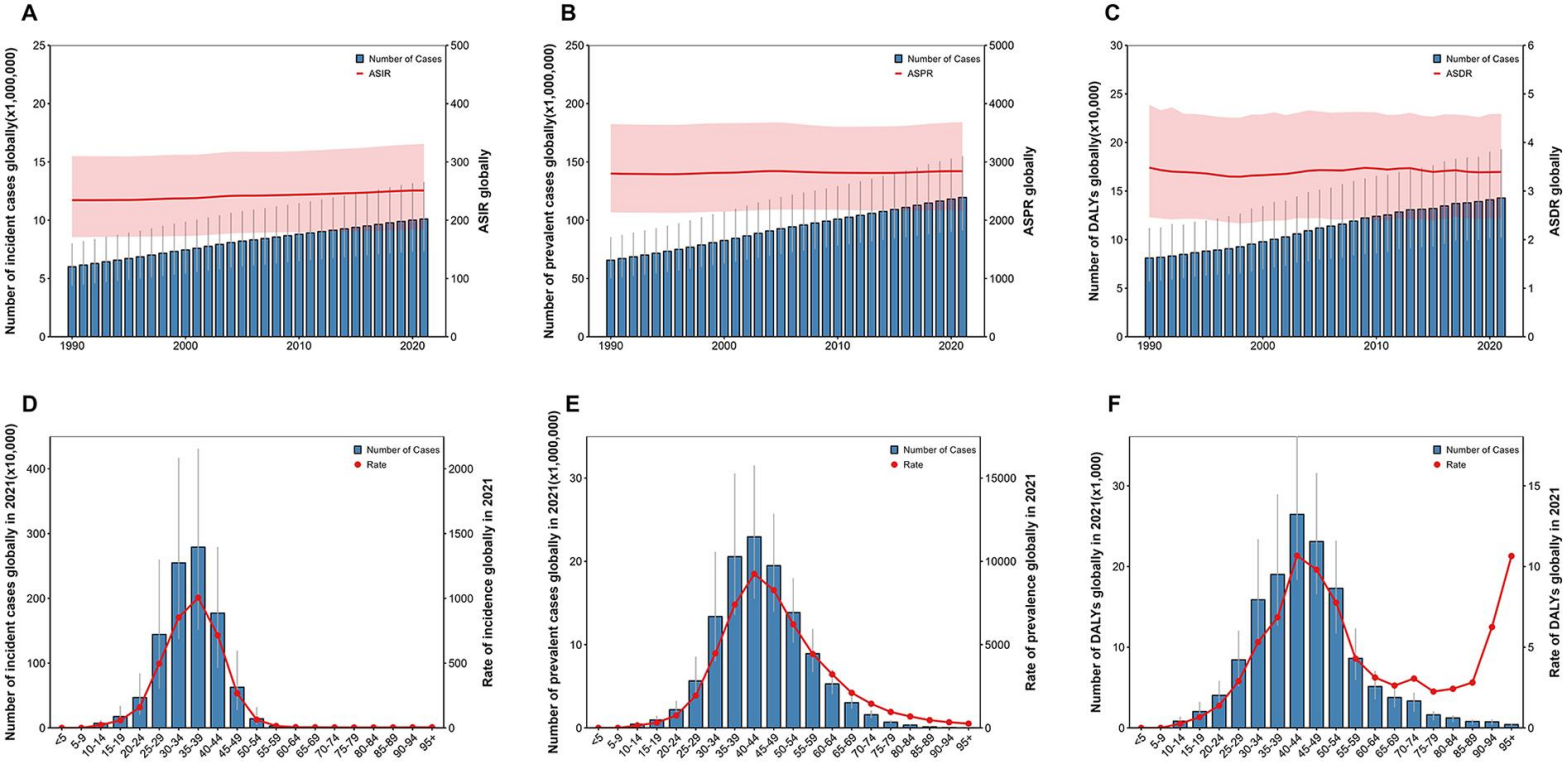
Global burden of uterine fibroids (UF) from 1990 to 2021. **(A)** The number of incident cases and age-standardized incidence rate (ASIR) globally. **(B)** The number of prevalent cases and age-standardized prevalence rate (ASPR) globally. **(C)** The number of disability-adjusted life years (DALYs) and age-standardized DALY rate (ASDR) globally. **(D)** Age distribution of incident cases globally in 2021, with the rate of incidence by age group. **(E)** Age distribution of prevalent cases globally in 2021, with the rate of prevalence by age group. **(F)** Age distribution of DALYs globally in 2021, with the rate of DALYs by age group.

Globally, the number of new cases of UF increased from 6,009,553 (95%UI: 4,390,470–8,011,360) in 1990 to 10,100,271 (7,350,444–13,285,677) in 2021.The ASIR for UF increased from 234.36 (171.06–309.92) per 100,000 in 1990 to 250.93 (183.44–330.94) per 100,000 in 2021. From 1990 to 2021, the ASIR increased at an EAPC of 0.24 (0.23–0.25) per year (Table 1). Globally, both the number of new UF cases and the ASIR rose from 1990 to 2021 (Figure 1). Prevalent UF cases increased from 65,694,590 (95% CI: 50,022,243–85,557,561) in 1990 to 119,544,904 (91,228,328–154,944,149) in 2021. The ASPR for UF in 1990 was 2,799.88 (2,133.46–3,650.54) per 100,000, and in 2021, it was 2,841.07 (2,164.43–3,682.27) per 100,000. From 1990 to 2021, the EAPC for ASPR was 0.04 (0.03–0.06). Globally, from 1990 to 2021, both the prevalence of UF and ASPR rose (Figure 1). The DALYs for UF increased from 81,142 (57,125–111,989) in 1990 to 142,885 (102,183–192,988) in 2021. The ASDR remained relatively stable, changing from 3.48 (2.46–4.77) per 100,000 in 1990 to 3.39 (2.43–4.59) per 100,000 in 2021, with an EAPC of 0.05 (−0.00, 0.11), indicating no significant statistical change. Globally, from 1990 to 2021, both the prevalence of UF cases and the associated DALYs rose (Figure 1).

**Table 1:** Incidence and ASIR of UF in 1990 and 2021 and the temporal trends from 1990 to 2021.

Location	Number of incident cases, 1990	ASIR per 100,000 population, 1990	Number of incident cases, 2021	ASIR per 100,000 population, 2021	EAPC of ASIR, 1990–2021
Global	6,009,553 (4,390,470, 8,011,360)	234.36 (171.06, 309.92)	10,100,271 (7,350,444, 13,285,677)	250.93 (183.44, 330.94)	0.24 (0.23, 0.25)
Low SDI	448,649 (324,083, 602,294)	221.80 (160.21, 296.73)	1,182,921 (858,970, 1,588,035)	237.87 (171.82, 315.92)	0.28 (0.25, 0.31)
Low-middle SDI	1,114,718 (812,004, 1,492,492)	223.03 (162.64, 297.72)	2,608,642 (1,905,584, 3,489,706)	262.69 (191.79, 350.05)	0.65 (0.59, 0.72)
Middle SDI	1,643,798 (1,195,917, 2,200,615)	197.65 (143.77, 263.13)	3,120,189 (2,273,529, 4,102,517)	238.21 (174.87, 313.82)	0.58 (0.55 ,0.61)
High-middle SDI	1,538,211 (1,113,593, 2,048,532)	277.83 (202.22, 366.07)	1,770,558 (1,294,484, 2,311,244)	256.86 (188.01, 334.87)	−0.29 (−0.34, −0.24)
High SDI	1,257,846 (922,701, 1,649,758)	262.24 (192.80, 342.64)	1,410,127 (1,017,456, 1,837,590)	265.52 (193.64, 348.39)	0.07 (−0.10, 0.23)
Central Asia	126,169 (91,127, 170,429)	411.92 (296.63, 547.76)	226,190 (162,305, 296,419)	436.75 (315.72, 568.20)	0.21 (0.19, 0.22)
Central Europe	169,096 (123,215, 222,683)	254.24 (186.78, 329.19)	152,371 (110,543, 197,355)	248.04 (185.24, 316.71)	−0.12 (−0.26, 0.03)
EasternEurope	739,222 (531,212, 979,789)	605.27 (441.11, 791.73)	705,091 (501,337, 932,122)	610.42 (445.44, 798.58)	0.07 (0.05, 0.09)
Australasia	9,849 (7,000, 12,964)	88.04 (62.91, 116.19)	14,101 (10,167, 18,832)	87.86 (63.79, 117.13)	−0.06 (−0.08, −0.04)
High-income Asia Pacific	251,480 (180,042, 339,316)	281.14 (200.23, 379.46)	217,245 (160,493, 281,971)	296.79 (219.27, 386.14)	0.15 (0.03, 0.27)
High-income North America	327,037 (242,148, 427,779)	203.45 (151.77, 263.43)	451,012 (319,517, 594,256)	252.30 (180.16, 335.13)	0.91 (0.54, 1.28)
Southern Latin America	63,593 (45,846, 88,040)	259.53 (187.13, 358.71)	100,931 (70,666, 135,009)	276.55 (193.62, 371.61)	0.11 (−0.00, 0.22)
Western Europe	712,609 (512,701, 949,248)	356.50 (258.03, 474.32)	711,356 (513,218, 936,554)	345.03 (250.25, 460.96)	−0.05 (−0.10, 0.00)
Andean Latin America	89,109 (65,231, 120,466)	512.27 (373.95, 686.87)	185,722 (135,694, 247,467)	521.33 (380.49, 694.99)	0.02 (−0.01, 0.04)
Caribbean	62,931 (45,749, 84,279)	361.08 (260.13, 480.93)	90,490 (64,956, 122,346)	367.58 (263.71, 497.19)	0.01 (−0.02, 0.05)
Central Latin America	337,250 (243,036, 453,351)	454.30 (325.84, 603.09)	620,704 (444,734, 815,811)	446.82 (319.89, 588.04)	−0.09 (−0.11, −0.08)
Tropical Latin America	139,034 (103,152, 181,603)	181.39 (137.14, 233.57)	363,754 (264,692, 476,322)	278.54 (204.44, 365.57)	1.40 (1.31, 1.50)
North Africa and Middle East	169,542 (122,300, 225,742)	118.25 (85.80, 158.20)	401,541 (289,713, 540,267)	122.58 (88.51, 163.47)	−0.02 (−0.06, 0.02)
South Asia	1,149,229 (836,887, 1,549,090)	243.08 (175.73, 326.15)	2,902,999 (2,098,361, 3,894,859)	296.47 (213.91, 395.25)	0.85 (0.77, 0.92)
East Asia	805,490 (574,969, 1,101,400)	125.52 (91.02, 170.75)	1,016,351 (735,440, 1,325,298)	136.50 (101.31, 177.06)	0.19 (0.07, 0.32)
Oceania	3,756 (2,688, 5,140)	129.49 (94.03, 175.69)	9,537 (6,856, 13,041)	139.06 (101.13, 189.15)	0.18 (0.15, 0.21)
Southeast Asia	311,435 (224,291, 416,987)	135.24 (98.92, 179.53)	543.289 (398.636, 718.133)	144.48 (106.40, 191.70)	0.18 (0.14, 0.21)
Central sub-Saharan Africa	56,612 (41,183, 75,933)	254.53 (184.18, 341.19)	159,378 (116,575, 210,784)	267.88 (195.21, 353.82)	0.17 (0.14, 0.19)
Eastern sub-Saharan Africa	153,295 (110,657, 205,801)	200.48 (145.14, 265.73)	397,942 (286,113, 537,147)	206.20 (149.22, 275.53)	0.12 (0.11, 0.13)
Southern sub-Saharan Africa	130,037 (95,385, 175,292)	532.06 (387.20, 710.22)	240,044 (175,190, 326,696)	534.10 (390.68, 720.81)	0.04 (0.01, 0.06)
Western sub-Saharan Africa	202,779 (146,821, 271,872)	261.40 (189.80, 348.89)	590,223 (430,423, 796,707)	272.75 (198.05, 364.30)	0.09 (0.06, 0.13)

ASIR, age-standardized incidence rate; EAPC, estimated annual percentage change; UF, uterine fibroids.

### 3.2 UF burden in 21 GBD regions

**Figure 2:**
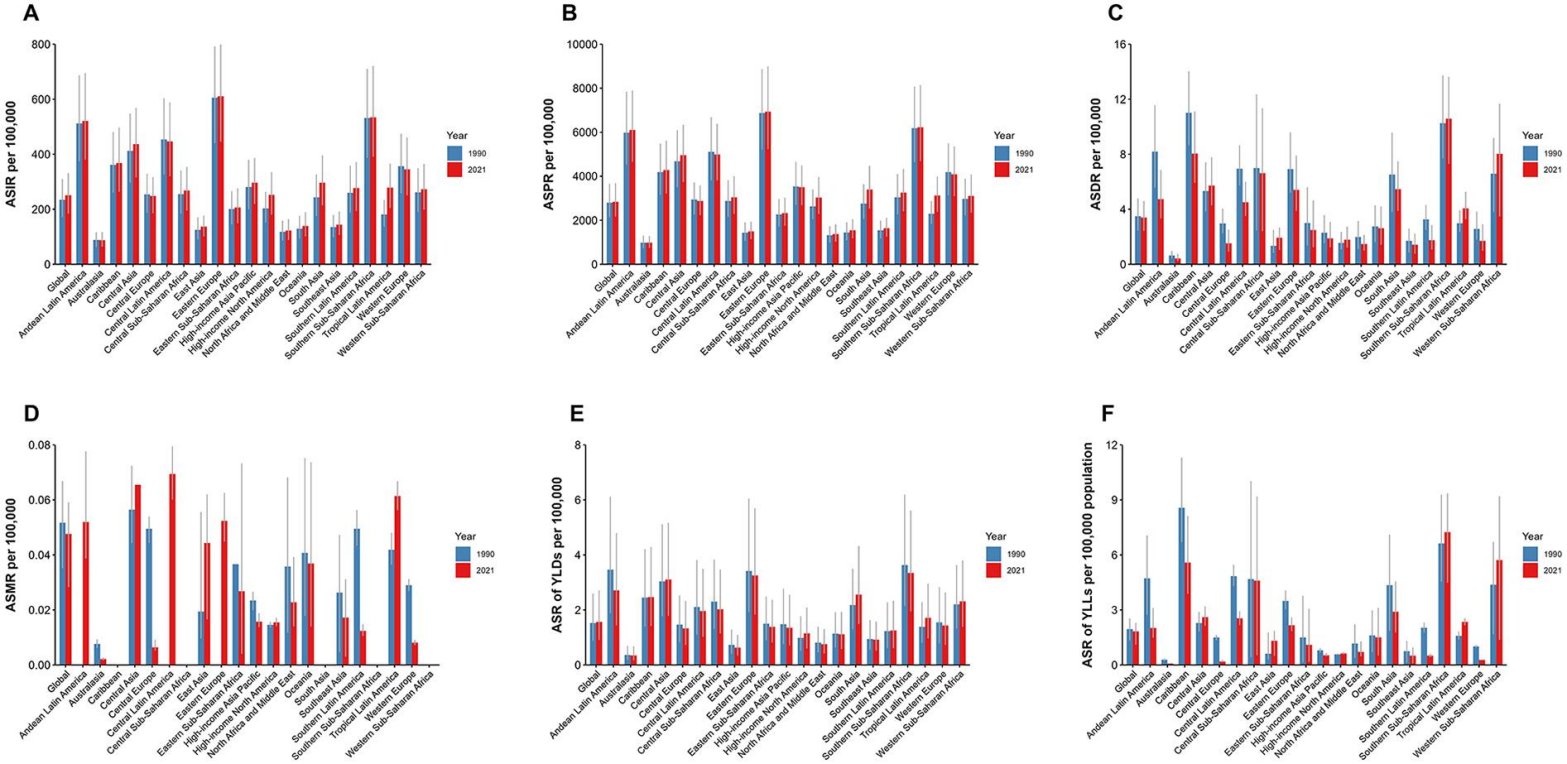
Global distribution of uterine fibroids (UF) burden across regions from 1990 to 2021. **(A)** Age-standardized incidence rate (ASIR) of UF by region, showing regional variations in the incidence over time. **(B)** Age-standardized prevalence rate (ASPR) of UF by region, highlighting differences in the prevalence across various regions globally. **(C)** Age-standardized DALY rate (ASDR) of UF by region, illustrating the impact of UF on health outcomes across different regions. **(D)** The rate of incidence of UF in 2021 by region, with a focus on the most affected regions. **(E)** The rate of prevalence of UF in 2021 by region, with regional variations in the prevalence rate. **(F)** The rate of DALYs from UF in 2021 by region, showing the burden of UF in terms of disability-adjusted life years across regions.

Between 1990 and 2021, 17 of the 21 Global Burden of Disease (GBD) regions experienced an increase in the age-standardized incidence rate (ASIR) of UF. In South Asia, new UF cases increased from 1,149,229 (95% UI: 836,887–1,549,090) in 1990 to 2,902,999 (2,098,361–3,894,859) in 2021. In 1990, Oceania reported the fewest new cases, totaling 3,756 (2,688–5,140), which increased to 9,537 (6,856–13,041) by 2021. Eastern Europe had the highest ASIR, with 605.27 (441.11–791.73) per 100,000 in 1990 and 610.42 (445.44–798.58) per 100,000 in 2021. Australasia reported the lowest ASIR, with 88.04 (62.91–116.19) per 100,000 in 1990 and 87.86 (63.79–117.13) per 100,000 in 2021. The highest EAPC for ASIR was observed in Tropical Latin America, with an annual change rate of 1.40 (1.31–1.50), while Central Europe had the lowest EAPC, at −0.12 (−0.26, 0.03) (Table 1; Figure 2A).

Between 1990 and 2021, 16 of the 21 GBD regions experienced an increase in the age-standardized prevalence rate (ASPR) of UF. In South Asia, prevalent cases increased from 11,277,758 (95% UI: 8,425,178–14,969,226) in 1990 to 31,026,201 (23,111,923–41,226,526) in 2021. In Oceania, prevalent cases increased from 34,952 (26,335–46,162) in 1990 to 94,680 (71,466–125,436) in 2021. Eastern Europe had the highest ASPR, with 6,873.77 (5,206.09–8,872.85) per 100,000 in 1990 and 6,934.45 (5,230.48–8,997.04) per 100,000 in 2021. Australasia recorded the lowest ASPR, with 982.11 (718.38–1,304.30) per 100,000 in 1990 and 979.30 (737.69–1,282.38) per 100,000 in 2021. The highest EAPC for ASPR was found in Tropical Latin America, with an annual change rate of 1.02 (0.93–1.11), while Central Europe had the lowest EAPC at −0.12 (−0.23, −0.01) (Figure 2B).

Between 1990 and 2021, the ASDR for UF declined in 15 of the 21 GBD regions. South Asia had the highest DALYs for UF, with 26,020 (95% UI: 15,393–38,286) in 1990 and 49,297 (34,836–68,044) in 2021. Oceania had the fewest DALYs in 1990, with 68 (38–105), while Australasia had the fewest DALYs in 2021, with 78 (46–141). The highest ASDR in 1990 was observed in the Caribbean, with 11.01 (8.65–14.03) per 100,000, while in 2021, the highest ASDR was found in Southern Sub-Saharan Africa, with 10.58 (7.30–13.62) per 100,000. Australasia had the lowest ASDR, with 0.64 (0.44–0.96) per 100,000 in 1990 and 0.42 (0.24–0.76) per 100,000 in 2021. East Asia had the highest EAPC for ASDR, with an annual change rate of 2.32 (1.77–2.87), while Central Europe had the lowest EAPC at −1.91 (−2.16, −1.65) (Figure 2C).

Detailed data for UF-related mortality, YLLs, YLDs, and other indicators for 1990 and 2021, along with EAPC data, can be found in Figures 2D–F.

### 3.3 UF burden in 204 GBD countries

**Figure 3:**
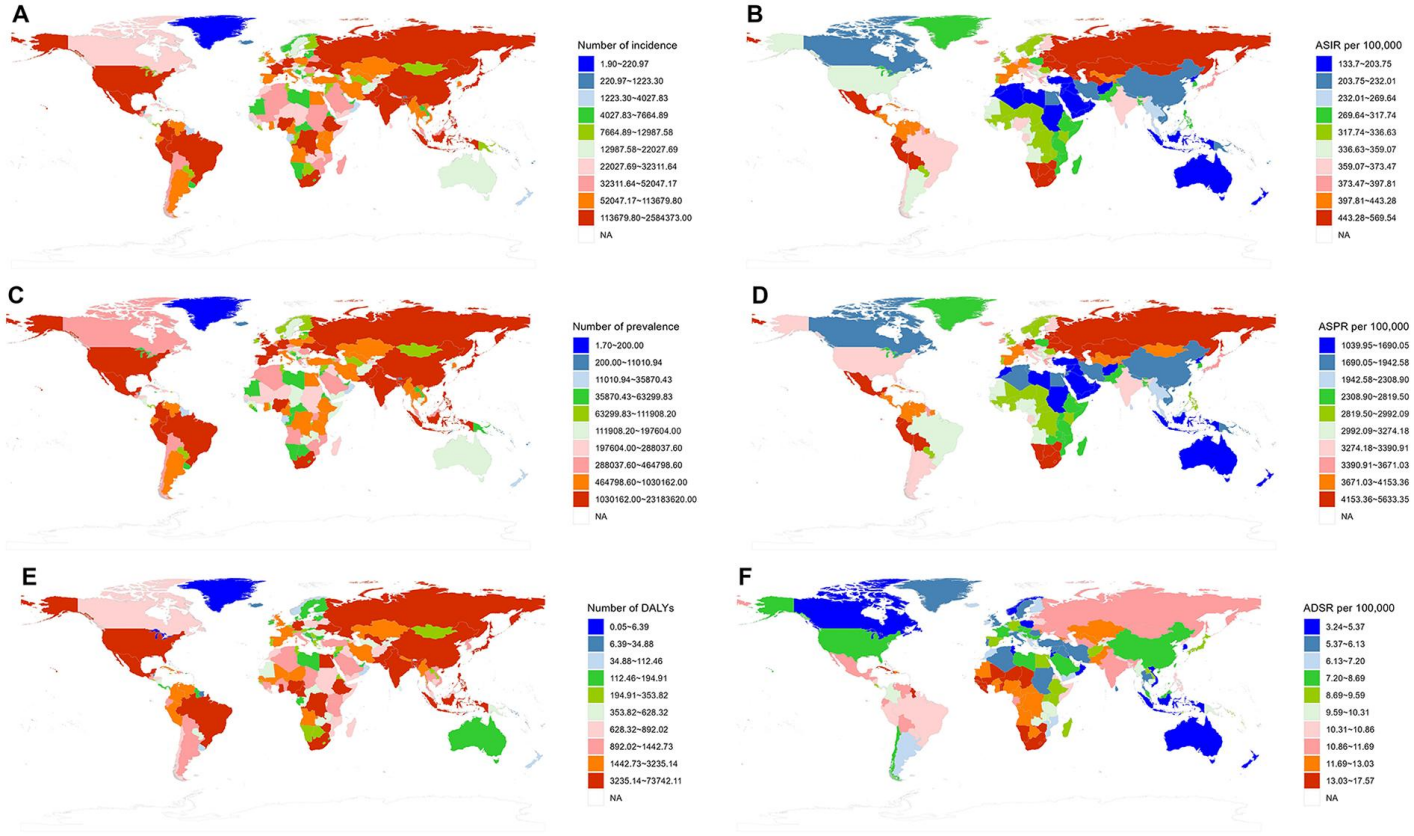
Global distribution of uterine fibroids (UF) burden by region in 2021. **(A)** The number of incident cases of UF by region. **(B)** Age-standardized incidence rate (ASIR) per 100,000 by region. **(C)** The number of prevalent cases of UF by region. **(D)** Age-standardized prevalence rate (ASPR) per 100,000 by region. **(E)** The number of DALYs due to UF by region. **(F)** Age-standardized DALY rate (ASDR) per 100,000 by region.

In 2021, the ASIR of UF showed significant international variation, with rates spanning from 83.69 to 686.95 per 100,000. Latvia, Russia, and Ukraine reported the highest incidence rates, with figures of 686.95 (95% UI: 480.42–918.98), 617.21 (453.11–804.78), and 604.21 (440.62–799.57) per 100,000, respectively. Conversely, New Zealand [83.69 (63.38–106.30) per 100,000], Australia [88.65 (63.68–119.63) per 100,000], and North Korea [108.25 (78.35–145.30) per 100,000] reported the lowest incidence rates. Between 1990 and 2019, Brazil [1.43 (1.33–1.53)], India [1.01 (0.88–1.14)], and the USA [0.96 (0.58–1.35)] exhibited the highest EAPC for ASIR. In contrast, the countries with the lowest EAPC for ASIR were Poland [−0.93 (−1.30, −0.56)], Iran [−0.73 (−1.06, −0.40)], and New Zealand [−0.64 (−0.71, −0.56)]. For further details and additional charts, please refer to the (Figure 3).

### 3.4 UF burden in SDI regions

**Figure 4:**
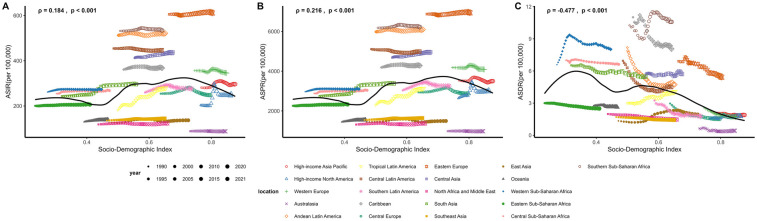
Association between socio-demographic index (SDI) and uterine fibroids (UF) burden across regions from 1990 to 2021. **(A)** Age-standardized incidence rate (ASIR) per 100,000 against SDI, with trends observed from 1990 to 2021. **(B)** Age-standardized prevalence rate (ASPR) per 100,000 against SDI, illustrating regional variations over time. **(C)** Age-standardized DALY rate (ASDR) per 100,000 against SDI, showing the correlation between SDI and UF-related health outcomes.

In 2021, the ASIR for UF varied among the five SDI groups: Low, Low-middle, Middle, High-middle, and High. The ASIR was highest in the High SDI group at 265.52 (95% UI: 193.64–348.39) per 100,000, while the Low SDI group had the lowest ASIR at 237.87 (171.82–315.92) per 100,000. Regionally, SDI positively correlated with ASIR (*ρ* = 0.184, *p* < 0.001). Both Tropical Latin America and Eastern Europe saw a continuous increase in ASIR from 1990 to 2021, which aligns with the increase in SDI (Table 1; Figure 4A). In 2021, the High SDI group had the highest ASPR of UF at 3,184.07 (2,450.98–4,074.76) per 100,000, while the Middle SDI group had the lowest at 2,600.71 (1,987.89–3,347.17) per 100,000. Regionally, SDI positively correlated with ASPR (*ρ* = 0.216, *p* < 0.001). Similar to the ASIR, both Tropical Latin America and Eastern Europe experienced an increase in ASPR from 1990 to 2021, consistent with the growth in SDI (Figure 4B). The ASDR was highest in the Low SDI group at 5.36 (3.09–7.56) per 100,000, while the High SDI group had the lowest rate at 1.69 (1.11–2.69) per 100,000. Regionally, SDI was negatively correlated with ASDR (*ρ* = −0.477, *p* < 0.001). Interestingly, despite the overall trend, the ASDR in Tropical Latin America continued to rise from 1990 to 2021, even as SDI increased, which contrasts with the broader regional trend (Figure 4C).

### 3.5 UF burden by age

According to global data from 2021, the burden of UF exhibits a clear age-related trend. Figure 1 presents data on incidence, prevalence, and DALYs categorized by age group. The global incidence of new UF cases reached its highest in the 35–39 age group in 2021 (Figure 1D). The incidence rate of UF notably rose in the 25–30 age group, peaking in the 35–39 age group. The incidence rate declined progressively after age 40, with a notably low rate in those over 50. In terms of prevalence (Figure 1E) and DALYs (Figure 1F), a similar trend was observed. As age increased, both the number and rate of prevalence and DALYs were highest in the 40–44 age group.

### 3.6 Health inequality analysis of UF burden

**Figure 5:**
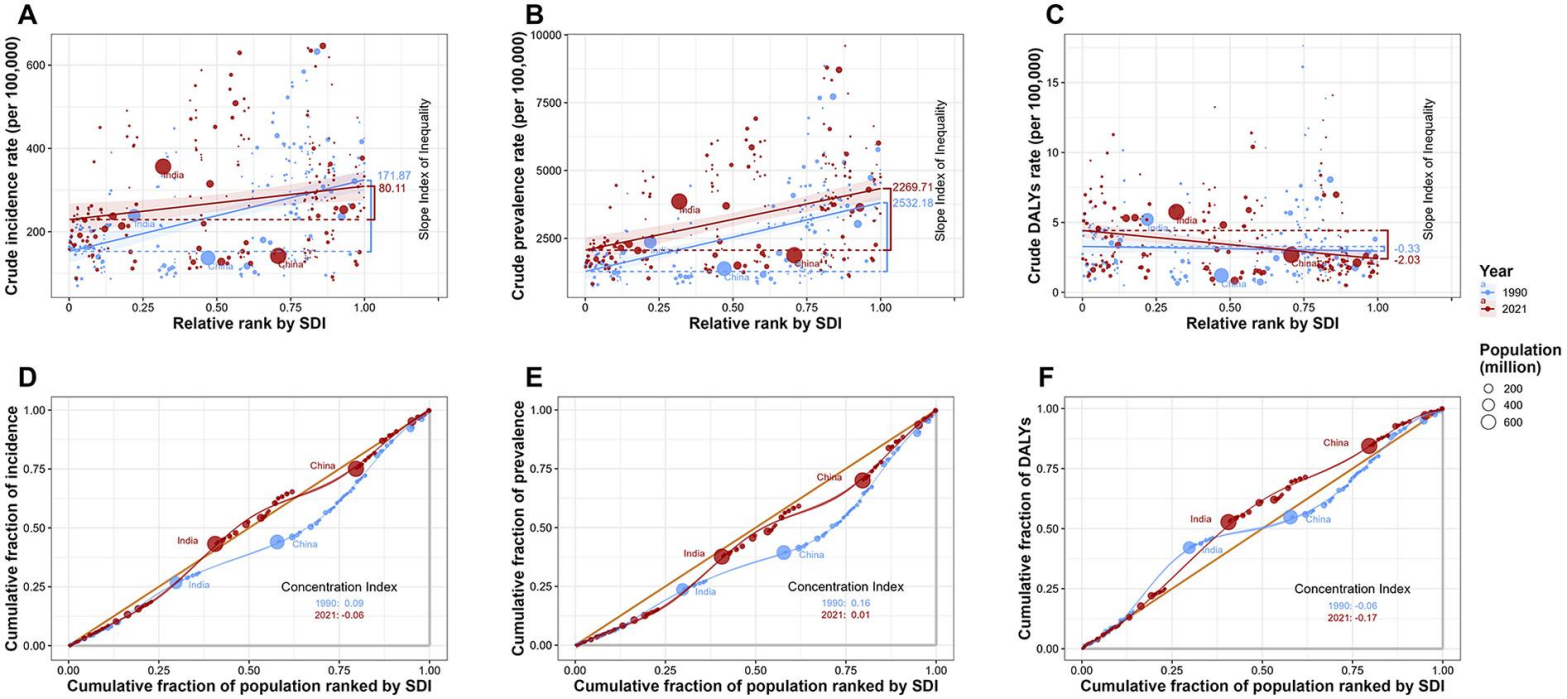
Health inequality analysis of uterine fibroids (UF) burden by socio-demographic index (SDI) from 1990 to 2021. **(A)** Crude incidence rate of UF per 100,000 against relative SDI rank, showing the slope index of inequality for 1990 and 2021. **(B)** Crude prevalence rate of UF per 100,000 against relative SDI rank, highlighting the shift in inequality over time. **(C)** Crude DALY rate of UF per 100,000 against relative SDI rank, with a focus on the changing health disparity from 1990 to 2021. **(D)** Cumulative fraction of incidence by SDI rank, with the concentration index for 1990 and 2021. **(E)** Cumulative fraction of prevalence by SDI rank, with the concentration index for 1990 and 2021. **(F)** Cumulative fraction of DALYs by SDI rank, with the concentration index for 1990 and 2021.

This study analyzed health inequalities associated with SDI and further identified trends in the changing burden of UF. In 1990, the SII for the incident of UF was 171.87, and for prevalent, it was 2,523.18, indicating that the burden of incident and prevalent was predominantly concentrated in high SDI countries. However, by 2021, the SII for the incident of UF decreased to 80.11, and for prevalent, it decreased to 2,269.71. This reduction in the SII for both incident and prevalent suggests that, while the burden of UF remains concentrated in high SDI countries, the level of health inequality has diminished. In 1990, the SII for UF's DALYs rate was −0.33, and by 2021, the SII for DALYs rate had increased to −2.03. This indicates that the DALY burden of UF is now predominantly concentrated in low SDI countries, with an increasing level of health inequality (Figures 5A–C). From 1990 to 2021, the concentration Index for UF also showed a tendency for the incidence and prevalence burden to gradually shift toward low SDI countries, along with a reduction in health inequality. However, the concentration index for DALYs remained negative and its absolute value increased, suggesting a growing trend in the DALY burden towards low SDI countries, with an increase in health inequality (1990 incident concentration index: 0.09, 2021 incident concentration index: −0.06; 1990 prevalent concentration index: 0.16, 2021 prevalent concentration index: 0.01; 1990 DALYs concentration index: −0.06, 2021 DALYs concentration index: −0.17) (Figures 5D–F).

### 3.7 Clustering analysis of UF burden

**Figure 6:**
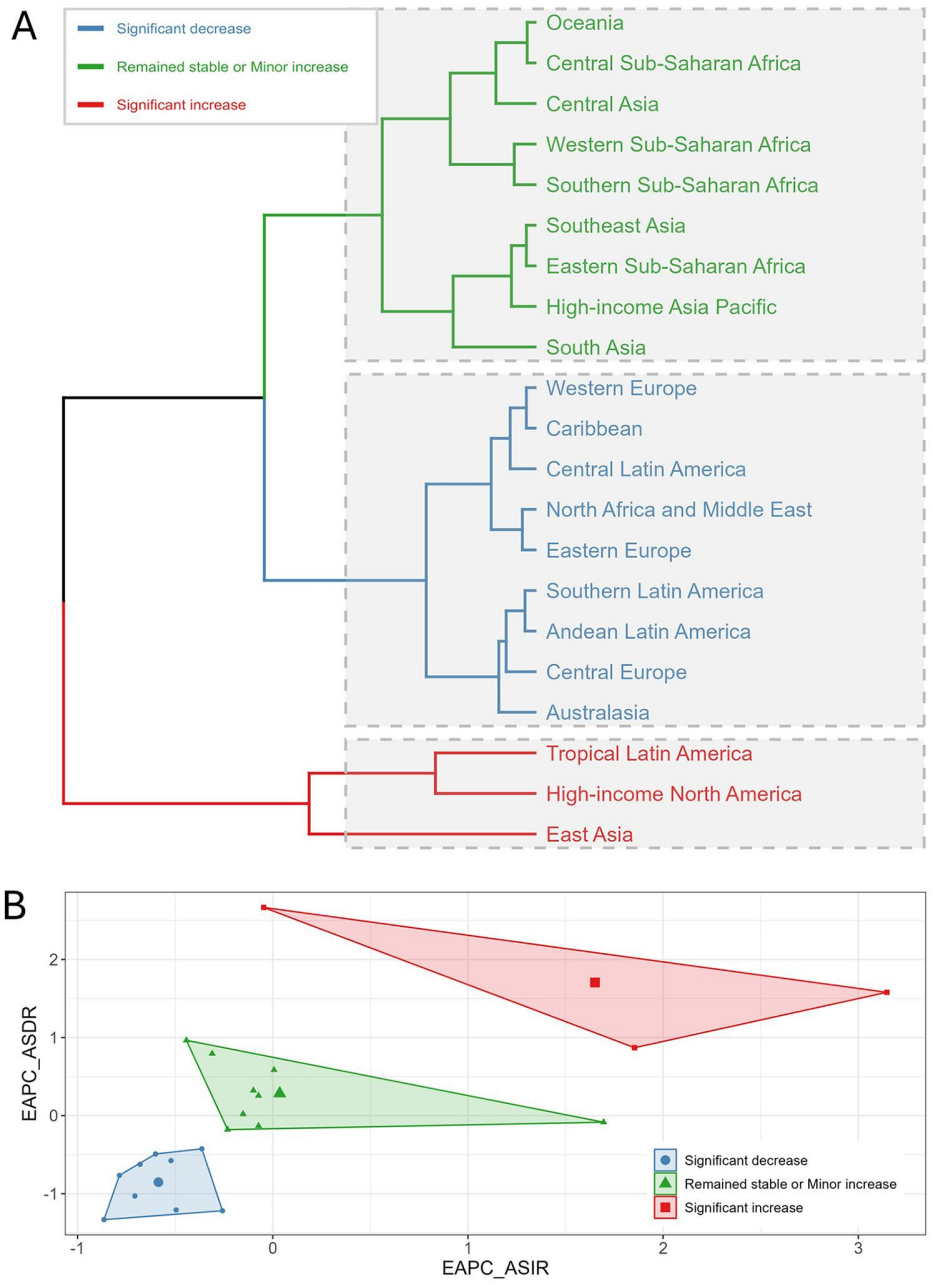
Clustering analysis of uterine fibroids (UF) burden by region from 1990 to 2021. **(A)** Hierarchical clustering of 21 Global Burden of Disease (GBD) regions based on trends in age-standardized incidence rate (ASIR) and age-standardized DALY rate (ASDR). Regions are categorized into three groups based on the magnitude of change in UF burden: significant decrease (blue), remained stable or minor increase (green), and significant increase (red). **(B)** Biplot of the estimated annual percentage change (EAPC) for ASIR and ASDR. Each region’s trend is represented with a marker indicating the direction and magnitude of change in both rates.

Based on the EAPC trends of ASIR and ASDR for UF from 1990 to 2021, we performed hierarchical clustering of 21 GBD regions, dividing them into three main clusters (Figures 6A,B). Regions highlighted in blue, such as Western Europe, the Caribbean, Central Latin America, North Africa and the Middle East, Eastern Europe, Southern Latin America, Andean Latin America, Central Europe, and Australasia, showed a notable decline in both ASIR and ASDR. These regions show a clear reduction in UF burden, with significant declines in both ASIR and ASDR. Regions highlighted in green, such as Oceania, Central and Western Sub-Saharan Africa, Central Asia, Southern and Eastern Sub-Saharan Africa, Southeast Asia, High-income Asia Pacific, and South Asia, exhibited stable or slightly increased ASIR and ASDR. According to the clustering results, these regions showed relatively small changes in ASIR and only slight fluctuations in ASDR. Regions highlighted in red, such as Tropical Latin America, High-income North America, and East Asia, showed notable rises in both ASIR and ASDR.

### 3.8 BAPC prediction analysis of UF burden

**Figure 7:**
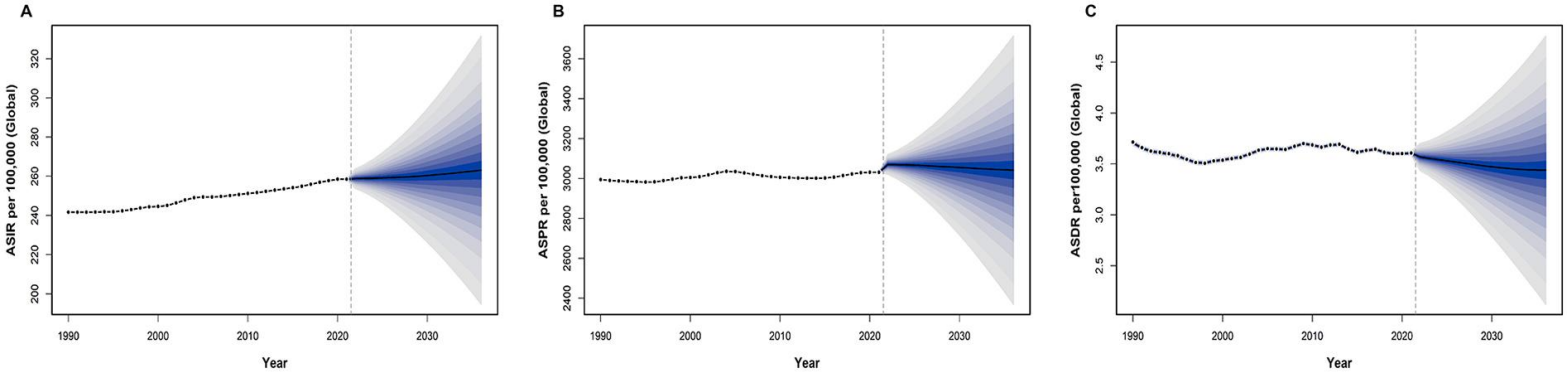
Projection of uterine fibroids (UF) burden from 2021 to 2036. **(A)** Projected global age-standardized incidence rate (ASIR) per 100,000 from 2021 to 2036, showing the expected trend and uncertainty interval. **(B)** Projected global age-standardized prevalence rate (ASPR) per 100,000 from 2021 to 2036, indicating future trends and uncertainty bounds. **(C)** Projected global age-standardized DALY rate (ASDR) per 100,000 from 2021 to 2036, demonstrating the expected trajectory of UF-related health outcomes.

Utilizing the BAPC package in R, we projected 15-year trends for ASIR, ASPR, and ASDR of UF. In 1990, the global ASIR for UF was 241.63 per 100,000. Despite fluctuations across the years, the overall trend shows a gradual increase in ASIR over time. By 2021, the ASIR rose to 258.53 per 100,000 and is projected to further increase to 263.19 per 100,000 by 2036 (Figure 7A). This alteration could indicate advancements in the early detection and surveillance of UF. The ASPR in 1990 was 2,995.07 per 100,000, showing minimal overall change with fluctuations around 3,000. In 2021, the ASPR was 3,031.75 per 100,000, and it is expected to remain stable, reaching 3,042.63 per 100,000 by 2036 (Figure 7B). Regarding ASDR, the rate in 1990 was 3.71 per 100,000, and mortality has declined each year since then. By 2021, the ASDR had decreased to 3.61 per 100,000, and this downward trend is expected to continue, with a projected ASDR of 3.44 per 100,000 in 2036 (Figure 7C).

These comparisons reveal the trends in ASIR, ASPR, and ASDR for UF throughout the prediction period. Figure 7A shows a continuous upward trend in ASIR, Figure 7B reflects minimal upward changes in ASPR, which remains stable, and Figure 7C indicates a steady decline in ASDR. Overall, while the ASDR burden of UF is on a downward trajectory, the rise in ASIR and ASPR remains a key concern for future monitoring.

## 4 Discussion

UF, the most prevalent benign tumors in the female reproductive system, have emerged as a major global health concern for women (3). Utilizing the 2021 Global Burden of Disease data, this study thoroughly examines the global, regional, and national trends in incidence, prevalence, and DALYs of UF from 1990 to 2021. In addition, the study includes an inequality analysis, clustering analysis, and BAPC predictions, aiming to provide valuable insights for future public health policies.

The study found that from 1990 to 2021, the global incidence, prevalence, and DALYs of UF significantly increased, while the ASIR and ASPR showed a slight upward trend, with EAPCs of 0.24% and 0.04%, respectively, and the ASDR remained stable without significant change.

It is crucial to differentiate the drivers behind the observed trends. The dramatic increase in the absolute number of incidence, prevalence and DALYs is largely attributable to demographic factors, specifically global population growth and the aging of the female population. In contrast, the modest but steady increase in the ASIR and ASPR suggests a true, albeit small, rise in the per-capita risk of developing or being diagnosed with UF over time. This epidemiological shift may be linked to changing risk factor profiles, such as race, genetic predisposition, lifestyle, rising obesity rates, hypertension and shifts in reproductive patterns, as well as improved detection and diagnosis in many parts of the world (22). Conflating these two trends could lead to misinterpretation; the primary public health challenge is the growing absolute number of women affected, which strains healthcare resources, while the slight increase in standardized rates points to underlying etiological or diagnostic factors that warrant further investigation.

Placing our findings in the context of other GBD-based gynecological disease studies offers valuable insights. For instance, the global burden of cervical cancer has shown a decreasing trend in age-standardized rates, largely due to successful screening programs and HPV vaccination, particularly in high-SDI countries (23). In contrast, the UF burden has increased, highlighting a different public health narrative. While cervical cancer represents a preventable infectious disease, UF is a non-malignant condition influenced by hormonal, genetic, and lifestyle factors. The comparison underscores that as countries undergo epidemiological transition and successfully combat infectious and vaccine-preventable diseases, the relative burden of non-communicable and chronic conditions like UF is likely to grow, demanding a shift in health policy and resource allocation. Unlike the trends for some malignancies such as ovarian cancer, which have shown stable or slightly decreasing rates in some high-income regions, the UF burden continues its upward trajectory, reinforcing its growing importance as a public health issue.

Socio-economic and cultural factors, such as education level and healthcare access, also play a crucial role. A study conducted in Haiti found that a higher level of education was associated with a lower prevalence of uterine fibroids, suggesting that education may reduce the incidence of uterine fibroids by improving health awareness and promoting early diagnosis (24). Conversely, increased healthcare access and awareness in some regions may lead to higher diagnostic rates, contributing to the observed rise in incidence. Treatment advancements also shape prevalence trends. While hysterectomy was once the primary treatment, less invasive options like uterine artery embolization and pharmacological therapies are now more common (25, 26). These uterus-preserving treatments may increase prevalence over time as more women are managed medically rather than surgically cured.

Although advancements in disease management and treatment may have contributed to the relatively stable trend in ASDR, they have not substantially decreased the incidence and prevalence of UF. Differences in healthcare resources, the availability of treatment options, and the coverage of early diagnosis can significantly impact the assessment of the disease burden of uterine fibroids, particularly in terms of DALYs, across different countries and regions. In high-income countries, where healthcare resources are abundant, the treatment of uterine fibroids is typically more standardized and aggressive, with a wider range of both surgical and non-surgical treatment options available. As a result, early diagnosis and treatment may help reduce the disease burden and decrease DALYs. Hysterectomy remains the traditional surgical approach for treating uterine fibroids, but in recent years, myomectomy as a uterus-preserving option, has gained increasing attention (27). Before the widespread adoption of laparoscopic techniques, abdominal surgery was the primary surgical approach for uterine fibroids. However, with advancements in surgical methods, laparoscopic surgery has gradually replaced open abdominal surgery for uterine fibroid procedures. Minimally invasive interventional and pharmacological therapies have also undergone substantial advancements in the management of UF. These advancements in uterine fibroid treatments contribute to a reduction in the DALYs burden associated with the condition. The increasing incidence and prevalent trends still present a significant challenge to global women's health.

At the regional level, the burden of UF shows significant heterogeneity. South Asia leads the world in both the incidence and prevalence of UF, likely due to its large population, uneven healthcare resource distribution, and socio-economic disparities (28). In contrast, regions such as Oceania and Australia have a relatively low burden of UF, likely due to factors such as lower population density, higher healthcare standards, and more developed social security systems (29). Our findings align with local studies; for instance, the high burden in regions with large populations of African descent is consistent with US-based studies showing higher prevalence in Black women, while the low burden in Australasia is consistent with regional estimates (2, 3, 19). These comparisons help validate the GBD estimates while highlighting the need for context-specific research.

However, it is important to note that epidemiological data on uterine fibroids are limited in certain regions, especially in low- and middle-income countries such as those in Africa, where the diagnosis rate is lower. This is partly due to the lack of typical symptoms in many women and limited opportunities for effective disease screening, which results in a significant number of undiagnosed cases (30, 31). Additionally, the lack of high-quality epidemiological data in these regions leads to insufficient awareness and management of the disease, further contributing to the underestimation of the global burden of uterine fibroids (32). This incomplete and poor-quality data particularly affects our understanding of the epidemiological variation of uterine fibroids across different ethnic groups and geographical regions. These findings may underestimate the true burden of uterine fibroids in low- and middle-income countries, with potential negative implications for policy and resource allocation. Therefore, future research should focus on improving diagnostic rates in these regions and encourage the enhancement of epidemiological data collection to better assess the global and regional burden of uterine fibroids.

The hierarchical clustering analysis of the EAPC for ASIR and ASDR of UF from 1990 to 2021 revealed three primary clusters among the 21 GBD regions. Regions indicated in red text, including Tropical Latin America, High-income North America, and East Asia, exhibited a notable increase in both ASIR and ASDR. Particularly, the Tropical Latin America region exhibited a rapid increase in the UF burden. Recent epidemiological studies in Latin America and the Caribbean indicate a notable increase in chronic and non-communicable diseases, attributed to rapid urbanization, lifestyle changes, and enhanced healthcare access (33, 34). The rise in chronic conditions like obesity and hypertension may affect UF incidence, as these factors are linked to a higher UF risk. Furthermore, as the region's public health system improves and the coverage of health screenings expands, early screening and diagnosis rates for UF have significantly increased, which may have led to the earlier detection and reporting of more cases, thus partly explaining the higher EAPC. The upward trends in High-income North America and East Asia are likely linked to advancements in medical technology and public health management improvements (35, 36). The significant enhancement of early screening and diagnostic capabilities in these regions has led to the detection and inclusion of more cases in the statistics.

National variations in ASIR of UF highlight significant healthcare disparities. Latvia, Russia, and Ukraine have the highest ASIR for UF, while New Zealand, Australia, and North Korea have the lowest ASIR. These differences may be attributed to variations in healthcare systems, health screening coverage, lifestyle factors, and socio-cultural influences across countries. For instance, some countries may diagnose UF earlier due to more extensive health screening programs, whereas others may underestimate the incidence of UF due to a lack of sufficient medical resources. Additionally, differences in global health policies and healthcare services may contribute to varying incidence rates of UF. Some countries may allocate more resources to early screening and diagnosis, leading to a higher reported incidence, while others may underreport actual incidence due to resource constraints (16). These international disparities highlight the importance of considering region-specific factors when developing health policies to effectively address the global burden of UF.

This study's health inequality analysis uncovered the link between socio-economic status and the burden of UF. The SII and concentration index reveal a shift in the incidence and prevalence of UF from high SDI countries in 1990 to middle- and low-SDI countries by 2021. This shift may be linked to changes in the global socio-economic structure, the reallocation of healthcare resources, and an increase in health awareness, particularly the improvement in diagnostic rates and the expansion of data coverage in low- and middle-SDI countries.

High-SDI countries typically have more advanced healthcare systems and greater healthcare accessibility, which means they can more effectively carry out early diagnosis and disease management. As a result, diagnostic rates for uterine fibroids in these countries are generally higher, leading to more accurate reporting of the disease burden. However, in low-SDI countries, although the actual burden of uterine fibroids may be similar to that in high-SDI countries, lower levels of healthcare and the lack of early diagnostic opportunities may result in many cases going undiagnosed or unreported. With advancements in diagnostic technology and improvements in healthcare resources, more low-SDI countries are beginning to identify and report more cases, which makes the disease burden appear to increase in these regions. Therefore, the observed shift in the burden of uterine fibroids toward low-SDI countries may, in part, be due to improvements in diagnosis and data collection, rather than an actual increase in burden. Given that the trends between SDI and uterine fibroid burden may be influenced by data quality, diagnostic technology, and reporting systems, future research should further investigate the role of these factors in the estimation of disease burden.

The burden of UF, as measured by DALYs, is predominantly concentrated in low-SDI countries, where health inequality has worsened. Despite advancements in diagnosis and treatment in some regions, the impact on women's health remains disproportionately high in resource-limited areas (15). Addressing health disparities necessitates prioritizing the needs of middle- and low-SDI countries and regions in global and national strategies for UF prevention and management, thereby mitigating socio-economic health inequalities.

This study utilized the BAPC model to forecast the 15-year trends in ASIR, ASPR, and ASDR of UF. The results predict that although the ASIR and ASPR of UF are expected to continue increasing, the ASDR is likely to steadily decrease. This prediction reflects global advancements in early diagnosis and treatment of UF, particularly in non-surgical treatments and early interventions (37–39). However, the increasing trends in ASIR and ASPR warrant attention, as they suggest that the burden of UF will continue to rise, placing greater strain on women's health and socio-economic resources.

Future efforts to prevent and treat UF will face multiple challenges, with significant variation in prevalence across countries and regions. As the burden of UF shifts increasingly toward low-SDI countries, particularly in low- and middle-income nations, these regions struggle with limited healthcare resources, inadequate screening, and inefficient diagnostic and treatment protocols. This results in barriers to early diagnosis and treatment for women, exacerbating the disease burden. Furthermore, women in impoverished areas often lack the financial means to access necessary treatments, further amplifying the impact of UF. In high-income countries, while overall incidence has decreased, certain groups remain at higher risk. Additionally, lifestyle and reproductive changes—such as poor diet, lack of exercise, environmental pollution, and delayed childbearing—may alter the pathophysiology of UF, complicating prevention and treatment efforts (40–42). Addressing health disparities due to socio-economic factors worldwide, especially in middle- and low-SDI countries, will be essential for advancing the prevention and treatment of UF.

While this study did not conduct a formal economic analysis, the findings have significant financial implications. The rising prevalence of UF, particularly in LMICs, suggests a growing, often unmeasured, economic burden from direct medical costs and indirect costs like lost productivity. As noted, the annual cost in the US is estimated in the tens of billions of dollars (15). Extrapolating this to a global scale, especially in regions with limited resources, highlights an urgent need for cost-effectiveness research on prevention and treatment strategies to guide resource allocation.

While offering an in-depth analysis of the global burden of UF using the GBD database, this study has certain limitations. First, the GBD data's dependence on country-level reports and statistical modeling can result in data quality inconsistencies, particularly in low- and middle-income countries. This may lead to an underestimation of the true disease burden, especially for a condition like UF that is often asymptomatic. Second, this study did not include a formal sensitivity analysis to test the robustness of our findings against different modeling assumptions, which limits the certainty of the conclusions. Third, the GBD dataset does not contain individual-level data on risk factors such as race, lifestyle, or environmental exposures, nor does it capture patient-reported outcomes (PROs) or the psychological impact of the disease. This means our analysis cannot explore the etiological drivers or the full patient experience of UF. Fourth, our predictions are based on the BAPC model, which assumes that past trends will continue; unforeseen changes in healthcare or risk factors could alter future outcomes. Finally, this analysis uses the GBD 2021 data, the most recent comprehensive dataset available. However, public health landscapes are dynamic, and continuous monitoring with updated data will be necessary to track future trends accurately.

This study examines GBD data from 1990 to 2021 to highlight the global, regional, and national burden and trends of UF. Despite the increasing global ASIR and ASPR of UF, the ASDR are projected to decline. However, there are significant disparities in the burden of UF between different regions and countries, and health inequalities persist. Future initiatives for preventing and treating UF should prioritize strengthening international collaboration, especially in middle- and low-SDI countries. Actionable steps include investing in low-cost diagnostic technologies, implementing targeted screening for high-risk populations, and developing public health campaigns to improve health literacy about UF. Additionally, future research should focus on region-specific risk factors, cost-effectiveness of interventions, and the patient experience to develop more effective prevention and treatment strategies, ultimately improving the health of women worldwide.

## Data Availability

The original contributions presented in the study are included in the article/Supplementary Material, further inquiries can be directed to the corresponding authors.
